# Sustainable Economic Development Through Entrepreneurship: A Study on Attitude, Opportunity Recognition, and Entrepreneurial Intention Among University Students in Malaysia

**DOI:** 10.3389/fpsyg.2022.866753

**Published:** 2022-04-07

**Authors:** Karina Wiramihardja, Varha N’dary, Abdullah Al Mamun, Uma Thevi Munikrishnan, Qing Yang, Anas A. Salamah, Naeem Hayat

**Affiliations:** ^1^Faculty of Business and Management, UCSI University, Kuala Lumpur, Malaysia; ^2^UKM – Graduate School of Business, Universiti Kebangsaan Malaysia, Bangi, Malaysia; ^3^College of Business Administration, Prince Sattam Bin Abdulaziz University, Al-Kharj, Saudi Arabia; ^4^Faculty of Entrepreneurship and Business, Universiti Malaysia Kelantan, Kota Bharu, Malaysia

**Keywords:** attitude towards entrepreneurship, opportunity recognition, entrepreneurial intention, university students, Malaysia

## Abstract

This study explored the effect of attitude towards entrepreneurship (ATE), need for achievement (NFA), risk-taking propensity (RTP), proactive personality (PRP), self-efficacy (SLE), opportunity recognition competency (ORC), entrepreneurship education, uncertainty avoidance (UNA), and entrepreneurial knowledge (ENK) on entrepreneurial intention (ENIN) among university students in Malaysia. This quantitative study had adopted the cross-sectional design approach and involved 391 university students in Malaysia via the online survey. The study outcomes revealed that the NFA, PRP, and SLE significantly affect students’ attitudes towards entrepreneurship. Moreover, entrepreneurship education and UNA significantly affect ORC. Finally, ATE has a positive and significant effect on ENIN among university students in Malaysia. As entrepreneurship offers an alternative career path for people seeking economic prosperity and addressing social issues, including unemployment, the government should formulate effective policies and regulations that support entrepreneurship activities. Universities and other institutions should play a pivotal role in providing the proper exposure via entrepreneurship education while honing the essential traits for a career in entrepreneurship.

## Introduction

Entrepreneurs have a crucial role in monetary exchanges and contribute to economic progress. Entrepreneurs are the engine of economic development due to their role in spurring the economy (Voda and Florea, 2019). Entrepreneurship practices differ across countries due to political, social, and economic variances (Naz et al., 2020). To date, some entrepreneurs are committed to improving the environment by driving innovative changes, efficiency, and better advancement (Osiri et al., 2019). Most university students are bound to join the labour force after graduation, and some students may turn into entrepreneurs and significantly contribute to Malaysia’s financial progress (Mahmood et al., 2019). In fact, the past decade has witnessed an exciting development within the entrepreneurial segment as undergraduate and postgraduate students displayed a keen interest in entrepreneurship (Kim et al., 2018).

The provision of employment opportunities is one of the fundamental aims of entrepreneurs, with entrepreneurship turning into a common vocation path (Schmitt et al., 2018). Entrepreneurship has become a compelling career choice amongst undergraduates worldwide (Voda and Florea, 2019), including Malaysia. The Malaysian government has initiated various undertakings to encourage involvement in innovative activities, especially among the youth, especially to support its financial development (Mahmood et al., 2020). The varying supporting structures and systems demonstrate the significance of entrepreneurship to the Malaysian economy.

As the global economy continues to shift, organisations need to continually rethink their business sectors, rebuild their activities, and alter their plans of action to remain competitive (Akhtar et al., 2020). Numerous countries worldwide, including Malaysia, have faced unemployment issues (Ahmad and Buchanan, 2015). Seeking a job is challenging as organisations progressively search for and retain skilled workers (Bagheri, 2017). The limited availability of jobs has forced the youth to compete for these positions. Unemployment rates continue to increase with the number of job seekers exceeding the job supply (Aloulou, 2016). Technology nurtures new business opportunities that only the knowledgeable and educated individual can indemnity and reap benefits (Kreuzer et al., 2021). The technology can help offer the right solution for the unemployed competing against one another for limited job openings (Hassan et al., 2020).

In Malaysia, the unemployed registered individual touches all time high 718,000 due to COVID-19 and later Movement control order (MCO). Whereas the unemployed graduate reaching the 200,000 limit due lack of economic activities and COVID-19 (Department of Statistics Malaysia, 2021). As a result, the Malaysian government has urged the youth to participate in entrepreneurial activities to increase the rate of Total Early-stage Entrepreneurial Activity (TEA) (Mahmood et al., 2020). Notably, the TEA rates are relatively low among those residing across the Asia Pacific and South Asia. Since Asians are more inclined to work as paid employees, it is pertinent to encourage them to be job creators instead of job seekers by unravelling their hidden potential to innovate to create financial and business values (Roxas, 2014). Entrepreneurs need to keep up with the rapidly evolving, varied, and competitive business climates. Entrepreneurial activities enhance human resources, which are critical in supporting a country’s economic progress (Li et al., 2020). Besides facing challenges and identifying opportunities, exposure to entrepreneurship may develop and hone one’s relationship-building abilities, authority, advancement, inventiveness, perseverance, and independence (Ploum et al., 2017). Equipping individuals with these entrepreneurial capacities and cultivating entrepreneurship can help boost financial development, apart from creating new ventures, wealth, and business opportunities (Newman et al., 2019). Entrepreneurial activities help achieve the government’s aspiration in bridging the gap between the poor and the rich, besides reducing people’s dependency on government assistance, which ultimately can reduce the burden on taxpayers (Wei et al., 2019).

Given the limited employment opportunities in light of the world financial crises, the Malaysian government has emphasised the need for graduates to be acquainted with entrepreneurship. The government hopes to increase the number of business entrepreneurs among university graduates, as this group possesses the capacity and knowledge to get involved in the business (Mahmood et al., 2020). Hence, it is imminent to ascertain entrepreneurial intention (ENIN) among graduates to realise the government’s aspiration. The present study assessed the factors that influenced ENIN among university students in Malaysia. Identifying the intentions of university students may facilitate the government in providing initiatives that promote entrepreneurial activities.

## Literature Review

### Theoretical Foundation

The conceptual framework applied in the current entrepreneurial study refers to the theory of planned behaviour (TPB) initiated by Ajzen (1991). Ajzen had introduced the TPB model to clarify how intention serves as a predictive factor for behaviour. The TPB upholds the notion that intention is influenced by three main components, namely: attitude towards entrepreneurship (ATE), subjective norm (SN), and perceived behavioural control (PBC), all of which should be adequate for the formation of intentions (Lortie and Castogiovanni, 2015). Nonetheless, Botsaris and Vamvaka (2016) asserted that the significance of each factor is relative and can fluctuate across behaviour, circumstance, and setting. The present study had adopted the TPB model in the Malaysian setting to determine the ENINs among Malaysian university students. It particularly concentrated on the effect of the TPB antecedents on intentions while considering entrepreneurship as an effective way to address the uprising unemployment issue.

### Entrepreneurial Intention

Entrepreneurial intention denotes the self-acknowledged confidence that one intends to start a new business with an established plan (Lortie and Castogiovanni, 2015). As starting a business is a deliberate and conscious decision, one’s intention is often the best indicator of entrepreneurial behaviour (Li et al., 2020). Ibrahim et al. (2017) noted that an entrepreneur must have the right attitude and motivation to prosper. This study had focussed on the effect of ATE and opportunity recognition competency (ORC) on the ENIN among university students in Malaysia.

### Predictors of Attitude Towards Entrepreneurship

Personal traits have been documented as one of the most significant predictors within the entrepreneurship domain (Mathieu and St-Jean, 2013). The need for achievement (NFA), risk-taking propensity (RTP), proactive personality (PRP), and self-efficacy (SLE) are integral personality traits that may affect one’s ATE, which can spark ENIN (Karabulut, 2016).

#### Need for Achievement

The NFA reflects the implication of the need to accomplish new and profound objectives consistently. The NFA is based on one’s expectation of performing better and faster than anybody else or their past performance (Akhtar et al., 2020). NFA spurs the desire to be a high performer, improve performance, strive for success, and feel responsible to learn continuously to succeed—denoting all the desirable characteristics to promote entrepreneurial activities (Ryan et al., 2011). NFA is one of the most common predictors of entrepreneurship (Ozaralli and Rivenburgh, 2016). It appears to be a common trait among entrepreneurs, with an empirical link between the NFA and entrepreneurial activities (Ryan et al., 2011). Those with a higher NFA possess a higher desire to be successful and recognised as successfully running a business. Hence, they would have a higher intention of becoming entrepreneurs to display their eagerness to succeed (Karabulut, 2016). Akhtar et al. (2020) reported that business founders tend to have higher NFA, a crucial element for a business to flourish. Thus, the following is proposed:

**Hypothesis (H1).** The NFA has a positive effect on the ATE

#### Risk-Taking Propensity

The concept of RTP refers to the extent of one’s willingness to take the risk when possible to incur loss (Norton and Moore, 2006). It means, one would still be willing to dedicate significant resources despite the possibility of losing them (Alvarez et al., 2006). Business visionaries have a higher propensity to accept risk and uncertainty upon venturing into new business endeavours (Ahmad and Xavier, 2012). RTP is a common trait among entrepreneurs, and entrepreneurs tend to take moderate to high risks compared to non-entrepreneurs (Alvarez et al., 2006). An earlier study reported a positive correlation between RTP and ENIN (Norton and Moore, 2006). Willingness to take risks has been associated with self-adequacy (Mahmood et al., 2020). Essentially, self-adequacy may affect one’s risk inclination, which later impacts the ENIN. Based on the explanation above, the second hypothesis of this study is as follows:

**Hypothesis (H2).** RTP has a positive effect on the ATE

#### Proactive Personality

Proactive people tend to take initiatives and readily identify opportunities, act on them, and persevere until they achieve their goals (Voda and Florea, 2019). People with PRP usually do not avoid problems but instead confront and solve problems (Newman et al., 2017). Being proactive aids one to adjust to changes and unexpected situations or environments (Li et al., 2020). By being proactive, one does not merely anticipate change; they initiate it for a more significant cause (Mathieu and St-Jean, 2013). In the business context, taking the initiative to improve the business is associated with being proactive as businesses continually need to deal with changes (Karabulut, 2016). Those with proactive behaviour are usually keen to take charge, initiate changes, and be flexible with role orientations (Voda and Florea, 2019), thus the higher possibility of becoming entrepreneurs (De Pillis and Reardon, 2007). Karabulut (2016) claimed that proactive People might have a higher intention of starting their own business than working for other people. Therefore, the following is hypothesised:

**Hypothesis (H3).** PRP has a positive effect on the ATE

#### Self-Efficacy

Within the entrepreneurial context, SLE refers to one’s confidence in his/her ability to perform entrepreneurial activities and functions (Dempsey and Jennings, 2014). Individuals with high SLE can pursue more challenging goals and persistently achieve those goals despite challenging circumstances. SLE is relevant within the entrepreneurship context, which often encounters high uncertainty and pressure (Piperopoulos and Dimov, 2015). SLE is a necessary entrepreneurship antecedent that predicts individuals’ intention to start new ventures, primarily due to their belief and confidence that they possess the capabilities to do so (Piperopoulos and Dimov, 2015). Entrepreneurs tend to possess high SLE (Newman et al., 2019), steadily strive through setbacks, and devise better strategies (Dempsey and Jennings, 2014). SLE influences start-up intentions, new venture growth, and business success by linking SLE with entrepreneurship. Akhtar et al. (2020) documented the significant influence of SLE on the intention to uptake entrepreneurial ventures. Thus, the following is proposed:

**Hypothesis (H4).** SLE has a positive effect on the ATE

### Predictors of Opportunity Recognition Competency

Wang et al. (2013) postulated that the individual competency in identifying opportunities is influenced by many factors—from individual to external environmental factors—within the entrepreneurial context. The following three key factors, entrepreneurship education (ENE), uncertainty avoidance (UNA), and entrepreneurial knowledge (ENK), were selected as determinants of ORC amidst university students in Malaysia.

#### Entrepreneurship Education

Entrepreneurship education refers to the procedures involved in imparting, developing, and shaping enterprising skills amongst undergraduates by improving the information gained from their experience and commitment in class (Wei et al., 2019). ENE and aptitudes developed throughout the study period via available courses usually empower the undergraduates’ inspiration and mentality from within to consider becoming entrepreneurs (Ahmad, 2013). ENE simultaneously improves students’ innovative skills and gives them enterprising inspirations (Del Rio-Rama et al., 2016). Besides, ENE can influence the students’ perspectives in facing the obstacles to starting a new business (Hassan et al., 2020). To date, universities have an active role in entrepreneurial development to commercialise the tertiary knowledge acquired by the students (Ibrahim et al., 2017). Several universities offer ENE to enhance ENIN among undergraduates, provide incubator offices, and mentor projects and systems (Ahmad and Buchanan, 2015). Entrepreneurship courses that increase students’ knowledge and aptitudes offer them business contacts and teach them about organisations and money-related assets, which are fundamental to perceive opportunities (Farani et al., 2017) and recognise viable opportunities (Ahmad and Buchanan, 2015). Hassan et al. (2020) postulated that the ENE influence opportunity recognition. Hence, the following hypothesis is proposed:

**Hypothesis (H5).** ENE has a positive effect on the ORC

#### Uncertainty Avoidance

Cultural and social norms vary across societies are created and moulded in differing environments, such as family, school, and association (Ozgen and Baron, 2007). UNA refers to a social characteristic that can be clarified by the extent of the society’s capacity to bear ambiguities and uncertainties (Mueller and Thomas, 2001). For some people, innovation and ambiguity are terrifying as both involve high-level uncertainty, which indicates high-level UNA (Schmitt et al., 2018). The appropriate flow of information with proper guidelines helps lower the level of uncertainty. Generally, disregard specific guidelines or rules unless necessary. Otherwise, they would not adhere to the guidelines as they would feel limited and suffocated (Şahin et al., 2019).

Since entrepreneurship is linked with uncertainty, it is imminent to adapt and develop themselves in uncertain conditions. Without uncertainty, entrepreneurship is unnecessary (Ukpere and Slabbert, 2009). Identifying opportunities is influenced by an entrepreneur’s insight and the capacity to see and manage uncertainty (Ozgen and Baron, 2007). In order to attain financial progress by causing changes and advancements, entrepreneurs must detect new opportunities, establish new ventures, and expand rivalry (Wang et al., 2013). Innovativeness, advancement, entrepreneurship, and creative mind are more critical than information-based activities (Wei et al., 2019). Şahin et al. (2019) claimed that entrepreneurship is positively correlated to development, opportunity recognition, and UNA. Taking the note from the above discussion following hypothesis was proposed:

**Hypothesis (H6).** UNA has a positive effect on the ORC

#### Entrepreneurial Knowledge

Entrepreneurial knowledge is crucial for one’s enterprising achievement and manageability (Botsaris and Vamvaka, 2016). ENK denotes the ideas, abilities, and attitudes that entrepreneurs utilise (Farani et al., 2017). Entrepreneurs with ENK have the “know what” about entrepreneurship (Ahmad and Buchanan, 2015). Equipping oneself with knowledge definitely affects an entrepreneur’s capacity to perceive and seek those opportunities (Hassan et al., 2020).

Entrepreneurs can understand, deduce, and apply the acquired knowledge in new exercises that are core to entrepreneurship (Piperopoulos and Dimov, 2015). Literature posited that the ENK comprises functional-orientated knowledge and strategic management-oriented knowledge (Farani et al., 2017). Functional-orientated knowledge incorporates marketing, deals, advertising, creation, human asset management, and budgetary management (Farani et al., 2017). On the other hand, strategic management-orientated knowledge includes knowledge of systems, critical investigations (e.g., overseeing the development, opportunity recognition, and use), business conditions evaluation (Botsaris and Vamvaka, 2016). The following hypothesis is proposed:

**Hypothesis (H7).** ENK has a positive effect on the ORC

### Attitude Towards Entrepreneurship

Attitude towards behaviour refers to one’s overall evaluation of performing the behaviour and the extent of the attractiveness (Botsaris and Vamvaka, 2016). In entrepreneurship, ATE denotes personal attraction and assesses the attractiveness of the entrepreneurship behaviour, positive or negative (Mahmood et al., 2020). The TPB upholds that ATE motivates people to have ENIN. ATE may be affected by intrinsic factors, which later influence one’s decision to become an entrepreneur (Ibrahim et al., 2017). Behavioural beliefs may determine ATE that individual gain beneficial outcomes from executing entrepreneurship behaviour (Botsaris and Vamvaka, 2016).

The entrepreneurship activity process is intentional and influenced by attitude and behaviour (Mahmood et al., 2020). ENIN and ATE have a constructive relationship, whereby one with an inspiring ATE is inclined to work independently (Ibrahim et al., 2017). Individuals with high-level TEA tend to favour self-employment than organisational employment, which signifies their intention to involve in entrepreneurship (Mahmood et al., 2019). Based on the explanation above, this study hypothesised the following:

**Hypothesis (H8).** ATE has a positive effect on the ENIN

### Opportunity Recognition Competency

Opportunity recognition competency refers to the ability to find openings—particularly in the business setting—and brainstorm innovative ideas to establish or grow a business (Guo et al., 2016). It also reflects entrepreneurial alertness (Bagheri, 2017). Identifying the right opportunity is vital for enabling start-up companies to multiply (Ozgen and Baron, 2007). The theory of resource-based view (RBV) emphasises the importance of the “right” resources to attain sustainable competitive advantage (Kreuzer et al., 2021). Both the ability and the competency to recognise opportunities are resources that only a few people possess (Kim et al., 2018).

People have varying personal attributes that contribute to their ability to recognise opportunities. Wang et al. (2013) postulated that personal attributes refer to prior knowledge, SLE, social networks, as well as perceptions of industrial and environmental opportunities. As opportunity recognition is necessary for entrepreneurship, entrepreneurs should implicitly be mindful of the need to perceive and exploit opportunities (Guo et al., 2016). The competency to detect the “right” opportunities for new ventures is integral, particularly for entrepreneurs to succeed (Bagheri, 2017). The following hypothesis is proposed:

**Hypothesis (H9).** ORC has a positive effect on the ENIN

## Research Methodology

The present study adopted a quantitative and cross-sectional design to gather data from university students in Malaysia aged 18 to above 30 years via an online survey. The cross-sectional research design is a quick and economical way for researchers to capture vast data (Podsakoff et al., 2003). Since the survey tool was developed from past studies and distributed to the target population using Google form, questionnaires were employed in this study. The current quantitative study sheds light on the variables that could influence ENIN, eventually influencing entrepreneurial behaviour.

The explanatory research approach was embraced to assess factors that determined the ENIN of university students in Malaysia. The collected data were analysed using the SmartPLS software, while descriptive statistics were applied to examine the determinants of the ENIN of university students in Malaysia. This study used G-Power 3.1 to determine the required sample size. Based on the power of 0.95 (social and behavioural science research requires a value above 0.80) and the effect size of 0.15, the ideal sample size should be at least 156 for a model with nine predictors (Faul et al., 2007). Data were collected from 391 university students via an online survey to hinder issues related to the small sample size.

### Survey Instrument

The survey instrument of this study is presented in Table 1. The five-point Likert scale (strongly disagree, disagree, neither agree nor disagree, agree and strongly agree) was used for this study to determine the ENIN among university undergraduates in Malaysia.

**TABLE 1:** Survey instrument.

Code	Questions	Source
NFA – Item 1	I am pleased when I can take on added job responsibilities	Mahmood et al. (2019)
NFA – Item 2	I like to set challenging goals for myself on the job	
NFA – Item 3	I enjoy situations at work where I am personally responsible for finding solutions to problems	
NFA – Item 4	I try very hard to improve on my past performance at work	
RTP – Item 1	I am not willing to take risks when choosing a work environment	Norton and Moore (2006)
RTP – Item 2	I prefer a low risk/high security work environment with predictable income over a high risk and high reward environment	
RTP – Item 3	I prefer to remain in an environment that has problems that I know about rather than to take the risks of a new environment that has unknown problems, even if the new environment offers greater rewards	
RTP – Item 4	I view job-related risk as a situation to be avoided at all costs	
RTP – Item 5	I don’t like to put something at risk, I would rather be on the safe side	
PRP – Item 1	I am constantly on the lookout for new ways to improve my life	Osiri et al. (2019)
PRP – Item 2	I can spot a good opportunity long before others can	
PRP – Item 3	I feel driven to make a difference in my community, and maybe the world	
PRP – Item 4	No matter what the odds, if I believe in something, I will make it happen	
PRP – Item 5	When I have a problem, I tackle it head-on	
SLE – Item 1	I can always manage to solve difficult problems if I try hard enough	Hassan et al. (2020)
SLE – Item 2	If someone opposes me, I can find the means and ways to get what I want	
SLE – Item 3	It is easy for me to stick to my aims and accomplish my goals	
SLE – Item 4	I am confident that I could deal efficiently with unexpected events	
SLE – Item 5	Thanks to my resourcefulness, I know how to handle unforeseen situations	
SLE – Item 6	I can solve most problems if I invest the necessary effort	
SLE – Item 7	I can remain calm when facing difficulties because I can rely on my coping abilities	
ENE – Item 1	My actual studies give me essential knowledge and tools to create my own company	Del Rio-Rama et al. (2016)
ENE – Item 2	Institutes would have to support company creation by students	
ENE – Item 3	I would like to have more subjects in my institute about entrepreneurship	
ENE – Item 4	The function to encourage entrepreneurship belongs to the institutes through education	
UNA – Item 1	If I had the opportunity and resources, I would love to start a business	Schmitt et al. (2018)
UNA – Item 2	My actual studies give me essential knowledge and tools to create my own company	
UNA – Item 3	Institutes would have to support company creation by students	
UNA – Item 4	I would like to have more subjects in my institute about entrepreneurship	
UNA – Item 5	The function to encourage entrepreneurship belongs to the institutes through education	
UNA – Item 6	Rules and regulations are important because they inform me what is expected of me	Roxas (2014)
ENK – Item 1	I have the knowledge required to start a business	
ENK – Item 2	The institute have helped me with getting the knowledge required to start a business	
ENK – Item 3	I have sufficient knowledge about specific training for young entrepreneurs	
ATE – Item 1	I feel uncomfortable when people ask me to do something and then do not give me the information I need to do it	Mahmood et al. (2019)
ATE – Item 2	It is important to have instructions spelled out in detail so that I always know what I am expected to do	
ATE – Item 3	I have the knowledge required to start a business	
ATE – Item 4	The institute have helped me with getting the knowledge required to start a business	
ATE – Item 5	I have sufficient knowledge about specific training for young entrepreneurs	
ORC – Item 1	“Seeing” potential new venture opportunities does not come very naturally to me	Ozgen and Baron (2007)
ORC – Item 2	While going about routine day-to-day activities, I see potential new venture ideas all around me	
ORC – Item 3	Recognising good opportunity usually requires experience in a specific industry or marketplace	
ORC – Item 4	Discussions with my family or friends can help me to recognise business opportunities	
ENIN – Item 1	I’m ready to make anything to be an entrepreneur	Hassan et al. (2020)
ENIN – Item 2	My professional goal is to be an entrepreneur	
ENIN – Item 3	I will make every effort to start and run my own firm	
ENIN – Item 4	I have seriously thought in starting a firm	
ENIN – Item 5	The institute have helped me with getting the knowledge required to start a business	
ENIN – Item 6	I’ve got the firm intention to start a firm one day	

*NFA, need for achievement; RTP, risk-taking propensity; PRP, proactive personality; SLE, self-efficacy; ENE, entrepreneurship education; UNA, uncertainty avoidance; ENK, entrepreneurial knowledge; ATE: attitude towards entrepreneurship; ORC, opportunity recognition competency; ENIN, entrepreneurial intention.*

### Common Method Variance

Harman’s (1976) one-factor test, which is the recommended test to address CMV (Podsakoff et al., 2003), was applied to evaluate the issue of CMV in this study. As a result, CMV did not emerge as a critical issue in this study, mainly because the main factor accounted for 28.75% of the variance, which is below the threshold value of 50% (Podsakoff et al., 2003).

### Multivariate Normality

Multivariate data normality was tested using Web Power,^1^ an online tool that verifies data normality, as prescribed by Peng and Lai (2012). The test results revealed that the dataset was not normal for the current dataset, as Mardia’s multivariate skewness (β = 12.43, *p-*value < 0.05) and kurtosis (β = 154.55, *p-*value < 0.05) *p*-values were below 0.05 (Cain et al., 2017).

### Data Analysis Method

The SmartPLS software was employed to analyse the quantitative data via information examination and statistical computations. Variance-based SEM, as suggested by Hair et al. (2019), was employed for this exploratory study with non-normality issues to explain changes in the dependent developments of the basic model.

## Data Analysis

### Demographic Characteristics

Table 2 presents the demographic characteristics of the respondents, which were composed of (32%) males and (68%) females. As for marital status, 96.7% of the respondents were single, 2.8% were married, while 0.3% were a divorcee and a widow. The respondents came from different study areas, as 46% of them pursued social sciences programmes, 35.3% from applied sciences programmes, 12.5% were enrolled in educational sciences programmes, and 6.1% undertook health sciences programmes. Regarding the average monthly household income, 44.5% of respondents came from households with incomes less than RM2,500, 23.8% respondents reported households with income ranging from RM2,501–RM5,000, 12.8% respondents were from households with incomes of RM5,001–RM7,500. 7.4% of respondents derived from households with incomes of RM7,501–RM10,000, 3.8% respondents came from households with RM10,001–RM12,500, and 7.7% respondents were from households with incomes exceeding RM12,500. Most respondents resided in urban areas (86.4%), while 13.6% of respondents lived in rural areas.

**TABLE 2:** Demographic characteristics.

	*N*	%		*N*	%
***Gender***			***Marital status***		
Female	266	68.0	Single	378	96.7
Male	125	32.0	Married	11	2.8
Total	391	100.0	Divorced	1	0.3
			Widowed	1	0.3
			Total	391	100.0
***Age group***			***Study area***		
18–20 years old	125	32.0	Social sciences	180	46.0
21–23 years old	203	51.9	Health sciences	24	6.1
24–26 years old	40	10.2	Educational sciences	49	12.5
27–29 years old	11	2.8	Applied sciences	138	35.3
>30 years old	12	3.1	Total	391	100.0
Total	391	100.0			
***Ethnicity***			***Occupation of household head***		
Malay	66	16.9	Private sector	126	32.2
Chinese	217	55.5	Public sector	44	11.3
Indian	22	5.6	Self-employment	82	21.0
Others	86	22.0	Unemployed	127	32.5
Total	391	100.0	Retired	12	3.1
			Total	391	100.0
***Average monthly household income***			***Living area***		
Less than RM2500	174	44.5	Urban	338	86.4
RM2,501–RM5,000	93	23.8	Rural	53	13.6
RM5,001–RM7,500	50	12.8	Total	391	100.0
RM7,501–RM10,000	29	7.4			
RM10,001–RM12,500	15	3.8			
More than RM12,500	30	7.7			
Total	391	100.0			

### Reliability and Validity

The mean and standard deviation scores for all variables exhibited that the respondents had a significant level of ENIN. Cronbach’s alpha (CA) was used to measure the reliability of the study constructs and strengthened with the values derived from Dillon-Goldstein’s (DG) rho and composite reliability (CR). Table 3 shows that all variables scored CA values above 0.7, thus confirming internal consistency and reliability. Besides, all variables had DG rho values above 0.7 and CR values above 0.8, which verified the reliability of all variables.

**TABLE 3:** Reliability and validity of variables.

Variables	No. items	Mean	SD	CA	DG *rho*	CR	AVE	VIF
NFA	4	4.070	0.625	0.759	0.782	0.846	0.580	1.705
RTP	5	3.473	0.855	0.833	1.138	0.856	0.547	1.039
PRP	5	3.910	0.646	0.786	0.795	0.853	0.538	2.157
SLE	7	3.739	0.682	0.857	0.871	0.891	0.539	2.083
ENE	4	3.746	0.733	0.709	0.719	0.819	0.531	1.687
UNA	6	4.161	0.595	0.824	0.830	0.873	0.535	1.182
ENK	3	3.260	0.963	0.823	0.889	0.892	0.733	1.533
ATE	5	3.746	0.911	0.918	0.926	0.939	0.754	1.091
ORC	4	3.865	0.671	0.711	0.718	0.820	0.534	1.091
ENIN	6	4.588	1.419	0.919	0.940	0.938	0.720	–

*NFA, need for achievement; RTP, risk-taking propensity; PRP, proactive personality; SLE, self-efficacy; ENE, entrepreneurship education; UNA, uncertainty avoidance; ENK, entrepreneurial knowledge; ATE, attitude towards Entrepreneurship; ORC, opportunity recognition competency; ENIN, entrepreneurial intention; SD, standard deviation; CA, Cronbach’s alpha; DG rho, Dillon-Goldstein’s rho; CR, composite reliability; AVE, average variance extracted; VIF, variance inflation factors. Source: Authors’ data analysis.*

The average value extracted (AVE) was employed to test the convergent validity of the variables. Ideally, AVE values above 0.5 are considered acceptable, and AVE values greater than 0.7 are deemed suitable (Chin, 2010). Table 3 shows that most of the variables had AVE values above 0.5. Hence, the AVE results demonstrated acceptable convergent validity for all the variables. The current study employed variance inflation factors (VIF) to assess multicollinearity issues. The VIF has been widely applied to eliminate multicollinearity (Hair et al., 2019). Table 3 shows that the VIF values of all variables had exceeded 1, but below 3.3. Thus, multicollinearity is not an issue for the current study.

Each study construct was assessed for discriminant validity based on the Fornell-Larcker criterion, Heterotrait–Monotrait (HTMT) Ratio, and loading, cross-loading table. The Fornell-Larcker criterion, discriminant validity, is established when the AVE of the associated latent construct exceeds the highest square correlation with another construct in the model. Table 4 displays that all constructs had fulfilled the criterion, indicating that discriminant validity is achieved with the Fornell-Laracker criterion. Next, the HTMT approach was used in this study to assess discriminant validity. Table 4 shows that all the HTMT values were below the threshold value of 0.9 (Henseler et al., 2014). Hence, discriminant validity was established for each study construct. The loading and the cross-loading values presented in Appendix 1. The result shows that the discriminant validity was established for the current study.

**TABLE 4:** Discriminant validity of constructs.

	NFA	RTP	PRP	SLE	ENE	UNA	ENK	ATE	ORC	ENIN
***Fornell-Larcker criterion***										
NFA	0.762									
RTP	0.093	0.739								
PRP	0.604	0.140	0.734							
SLE	0.574	0.193	0.686	0.734						
ENE	0.458	0.247	0.478	0.467	0.729					
UNA	0.369	0.233	0.332	0.351	0.390	0.731				
ENK	0.348	0.358	0.456	0.472	0.589	0.260	0.856			
ATE	0.395	0.142	0.445	0.457	0.631	0.232	0.454	0.869		
ORC	0.310	0.291	0.275	0.338	0.473	0.526	0.350	0.289	0.730	
ENIN	0.406	0.150	0.487	0.478	0.625	0.181	0.535	0.784	0.242	0.848
***Heterotrait-Monotrait ratio (HTMT)***										
NFA	–									
RTP	0.150	–								
PRP	0.785	0.145	–							
SLE	0.706	0.182	0.831	–						
ENE	0.625	0.285	0.646	0.601	–					
UNA	0.486	0.322	0.415	0.420	0.491	–				
ENK	0.436	0.359	0.571	0.568	0.762	0.308	–			
ATE	0.465	0.107	0.515	0.500	0.817	0.271	0.536	–		
ORC	0.422	0.444	0.354	0.426	0.630	0.679	0.432	0.342	–	
ENIN	0.484	0.143	0.571	0.532	0.815	0.213	0.672	0.837	0.301	–

*NFA, need for achievement; RTP, risk-taking propensity; PRP, proactive personality; SLE, self-efficacy; ENE, entrepreneurship education; UNA, uncertainty avoidance; ENK, entrepreneurial knowledge; ATE, attitude towards entrepreneurship; ORC, opportunity recognition competency; ENIN, entrepreneurial intention. Source: Authors’ data analysis.*

### Path Analysis

Path analysis enables researchers to evaluate the relationship between variables. Table 5 shows that the *r*^2^ value, which demonstrated that 25.6% of the variance in the attitude of university students towards entrepreneurship, was explained by their levels of NFA, RTP, PRP, and SLE. Next, the *Q*^2^ value of 0.187 indicated that university students’ NFA, RTP, PRP, and SLE had medium predictive relevance on their ATE.

**TABLE 5:** Path coefficients.

Hypo		Beta	CI - Min	CI - Max	*T*	*p*	*r* ^2^	*f* ^2^	Q^2^	Decision
***Factors affecting the attitude towards entrepreneurship***										
H_1_	NFA → ATE	0.139	0.033	0.257	2.011	0.022		0.015		Accept
H_2_	RTP → ATE	0.056	–0.099	0.148	0.873	0.192	0.256	0.004	0.187	Reject
H_3_	PRP → ATE	0.193	0.067	0.311	2.571	0.005		0.023		Accept
H_4_	SLE → ATE	0.234	0.122	0.344	3.278	0.001		0.035		Accept
***Factors Affecting Opportunity Recognition Competency***										
H_5_	ENE → ORC	0.262	0.157	0.372	3.923	0.000		0.064		Accept
H_6_	UNA → ORC	0.400	0.325	0.487	7.991	0.000	0.367	0.214	0.184	Accept
H_7_	ENK → ORC	0.091	–0.011	0.196	1.473	0.071		0.009		Reject
***Factor Affecting Entrepreneurial Intention among University Students***										
H_8_	ATE → ENIN	0.779	0.739	0.815	34.515	0.000	0.615	1.442	0.435	Accept
H_9_	ORC → ENIN	0.018	–0.043	0.074	0.492	0.311		0.001		Reject

*NFA, need for achievement; RTP, risk-taking propensity; PRP, proactive personality; SLE, self-efficacy; ENE, entrepreneurship education; UNA, uncertainty avoidance; ENK, entrepreneurial knowledge; ATE, attitude towards entrepreneurship; ORC, opportunity recognition competency; ENIN, entrepreneurial intention. Source: Authors’ data analysis.*

As presented in Table 5, the coefficient value for the effect of NFA on ATE (H_1_) was 0.139 with a *p*-value of 0.022. Thus, students’ NFA exerted a significantly positive effect on their ATE. The *f*^2^ value of 0.015 showed a small effect size that university students need to achieve on their ATE. The coefficient value for the effect of RTP on ATE (H_2_) was 0.056 with a *p*-value of 0.192. Hence, students’ RTP had an insignificant effect on their ATE. The *f*^2^ value of 0.004 depicted a small effect size, explaining that university students’ RTP did not predict their ATE.

The coefficient value for the effect of PRP on university students ATE (H_3_) was 0.193 with a *p*-value of 0.005. It showed that university students’ PRP exerted a significantly positive effect on their ATE. The *f*^2^ value of 0.023 indicated a small effect size of university students’ PRP in predicting their ATE. The coefficient value for the effect of SLE on university students ATE (H_4_) was 0.234 with a *p*-value of 0.001. Thus, the university students’ SLE was significantly positive on their ATE. The *f*^2^ value of 0.035 showed a small effect size of university students’ SLE in predicting their ATE.

As depicted in Table 5, the *r*^2^ value of 0.367 for university students’ ORC signified 36.7% of the variance in university students’ ORC, which can be explained by their ENE, UNA, and ENK. The *Q*^2^ value of 0.184 for university students’ ORC revealed a medium predictive relevance on ORC. The coefficient value for the effect of ENE on ORC (H_5_) was 0.262 with a *p*-value of 0.000, which signified a significantly positive correlation between the two variables. The *f*^2^ value of 0.064 indicated a small effect size of ENE in predicting university students’ ORC. The coefficient value for the effect of UNA on ORC (H_6_) was 0.400 with a *p*-value of 0.000. It revealed the significant positive impact of university students’ UNA on their opportunity recognition competence. The *f*^2^ value of 0.214 depicted a moderate effect size that enabled UNA to predict university students’ ORC.

The coefficient value for the effect of ENK on ORC (H_7_) was 0.091 with a *p*-value of 0.071. Hence, university students’ ENK had an insignificant effect on their ORC. The *f*^2^ value of 0.009 showed a small effect size, depicting that university students’ ENK could not predict their ORC. As shown in Table 5, the *r*^2^ value of 0.615 for university students’ ENIN indicated that their ATE and ORC could explain 61.5% of the variation in university students’ ENIN. The *Q*^2^ value of 0.435 signified that the university students’ ATE and ORC had a medium predictive relevance on their ENIN. The coefficient value for the effect of ATE on ENIN (H_8_) was 0.779 with a *p*-value of 0.000. Hence, university students’ ATE exerted a significantly positive effect on their ENIN. The *f*^2^ value of 1.442 showed a large effect size that revealed university students’ ATE could predict their ENIN. The coefficient value for the effect of ATE on ENIN (H_9_) was 0.018 with a *p*-value of 0.311. It signified an insignificant effect between university students’ ORC and their ENIN. The *f*^2^ value of 0.001 revealed a small effect size, which explained that university students’ ORC could not predict their ENIN.

### Mediating Effects

The study presents indirect effect coefficients, confidence intervals, and *p* values to determine the mediating effects of ATE and ORC (see Table 6). As a result, NFA, PRP, and SLE displayed a significantly positive and indirect effect on the ENIN of university students in Malaysia (*p*-value < 0.05). RTP exhibited an insignificantly indirect effect on ENIN (*p*-value > 0.05). It verified the mediating effect of attitude on entrepreneurship. For ORC, the statistically insignificant and indirect effect of ENE, UNA, and ENK on the ENIN of university students revealed that ORC had an impartial mediation effect on the correlations (*p*-value > 0.05).

**TABLE 6:** Mediating effects.

Associations	Beta	CI - Min	CI - Max	*T*	*p*	Decision
***Mediating effect of attitude towards entrepreneurship***						
NFA → ATE → ENIN	0.108	0.025	0.200	1.993	0.023	Accept
RTP → ATE → ENIN	0.044	–0.078	0.116	0.870	0.192	Reject
PRP → ATE → ENIN	0.150	0.052	0.248	2.552	0.006	Accept
SLE → ATE → ENIN	0.182	0.094	0.269	3.264	0.001	Accept
***Mediating effect of opportunity recognition competency***						
ENE → ORC → ENIN	0.005	–0.011	0.020	0.468	0.320	Reject
UNA → ORC → ENIN	0.007	–0.017	0.031	0.484	0.314	Reject
ENK → ORC → ENIN	0.002	–0.003	0.010	0.400	0.345	Reject

*NFA, need for achievement; RTP, risk-taking propensity; PRP, proactive personality; SLE, self-efficacy; ENE, entrepreneurship education; UNA, uncertainty avoidance; ENK, entrepreneurial knowledge; ATE, attitude towards entrepreneurship; ORC, opportunity recognition competency; ENIN, entrepreneurial intention. Source: Authors’ data analysis.*

## Discussion

The first hypothesis (H_1_) tested the correlation between university students’ NFA and ATE. The outcome revealed that students’ NFA displayed a significantly positive effect on their ATE. The current coincides with the finding posted by Akhtar et al. (2020) that the personal NFA harnesses the ATE. People with higher NFA show eagerness to become successful entrepreneurs (Karabulut, 2016). Next, the second hypothesis (H_2_) assessed the correlation between university students’ RTP and ATE. The analysis suggests that the RTP did not exert a statistically significant effect on ATE. The current finding contradicts the finding conveyed by Mahmood et al. (2020) that personal RTP instigates the ATE. Hence, the current finding suggests that the Malaysian students were unwilling to take risks, handle uncertainty, and assume personal risks to pursue entrepreneurship as a career. Following, the third hypothesis (H_3_) confirmed the positive and significant impact of PRP on the ATE. The outcome accord with the result posted by Voda and Florea (2019) that the PRP facilitates the individual to engage in change initiation and builds the necessary inclination to engage in entrepreneurship. Subsequently, the fourth hypothesis (H_4_) examined the effect of students’ SLE on the ATE. The result suggests that SLE significantly instigates the ATE among the study samples. The current finding agrees with the result postulated by Newman et al. (2019) that the SLE capacities harness the attitude to uptake entrepreneurship activities and empower people to perform better in organisational settings. Akhtar et al. (2020) stated that individual SLE instigates the individual inclination to engage in an entrepreneurship venture.

Following, the fifth hypothesis (H_5_) assessed the path between ENE and ORC to identify the impact of entrepreneurship education on the entrepreneurial capacity of opportunity recognition. As a result, ENE had a significantly positive impact on the ORC. The current outcome accord with finding suggested by Wei et al. (2019) that formal education empowers the student to identify the entrepreneurial opportunities that later instigates the entrepreneurial venture. Hassan et al. (2020) also identify that formal entrepreneurial education helps the Indian student’s capacity to formally research and realise the existing entrepreneurial opportunities or develop new business opportunities. Next, the sixth hypothesis (H_6_) examined the path between UNA and ORC to determine capabilities in bearing uncertainty that had affected the ability to recognise opportunities. The study institute that UNA exerted a significantly positive relationship with university students’ ORC. The present finding agrees with the outcome reported by Şahin et al. (2019) that UNA helps the opportunity recognition to develop the new business endeavour or engage in innovative or higher risk-taking business initiatives. Following, the seventh hypothesis (H_7_) explored the path between ENK and ORC to ascertain the effect of ENK on students’ capabilities in opportunities recognition. The study outcome disagrees with the finding posted by Farani et al. (2017) that the Malaysian students’ ENK did not build the student capacity to build, manage and grow the entrepreneurial ventures.

Next, the eighth hypothesis (H_8_) assessed the path between university students ATE and ENIN. As a result, the university students’ ATE displayed a significantly positive effect on ENIN. The finding is consistent with the outcome reported by Ibrahim et al. (2017) that the people with an ATE nurture the ENIN to engage in entrepreneurial ventures. Lastly, the hypothesis (H_9_) inspected the path between university students’ ORC and ENIN to determine. The outcome accord with the result documented by Bagheri (2017) that opportunity recognition is the integral attribute of an entrepreneurial mindset and facilitates the individual to form entrepreneurial ventures and work progressively.

The current study found that students ATE had a significant mediating effect in the relationship between the NFA and ENIN for the mediating analysis. The result depicts that the attitude facilitates the NFA on the ENIN. Next, the mediational effect of attitude between the PRP and entrepreneurial orientation was significant. The finding offers that the attitude significantly influences PRP’s effect on ENIN. Following, the significant mediational effect of attitude exists between SLE and ENIN. The finding suggests that the relationship between the RTP and ENIN is insignificantly mediated by ATE. It suggests that the risk-taking was not predicting the attitude for entrepreneurship, not instigating the ENIN.

However, the mediational effect of opportunity recognition between the ENE and ENIN was insignificant. The finding offers that the opportunity recognition did not transmit the effect of entrepreneurial education on the ENIN. Next, the insignificant mediational effect of ORC exists between UNA and ENIN. Lastly, the opportunity recognition also insignificantly mediates the relationship between ENK and entrepreneurship intention.

## Implications

The entrepreneurship literature elaborates the application of TPB as a framework in examining the effects of ATE, social norms, and PBC on ENIN and entrepreneurial behaviour (Mahmood et al., 2020). Nonetheless, only a handful of studies had evaluated the peripheral factors, such as ENE, ENK, and the ability to recognise opportunities among prospective entrepreneurs (Naz et al., 2020). The relationship between opportunity recognition and ENIN (Kreuzer et al., 2021). According to Lortie and Castogiovanni (2015), it is relatively unknown how opportunities recognition may fit into an entrepreneurial model through the lens of TPB.

The current study had assessed the effect of ATE (generated from NFA, RTP, PRP, and SLE) and ORC (generated from ENE, UNA, and ENK) harnessing the ENIN among Malaysian university students. Therefore, by examining new latent constructs that have been scarcely discussed, the current study offers new insight and enriches the entrepreneurship literature.

The study outcomes uphold the TPB model by demonstrating the positive effect of entrepreneurship attitude on students’ ENIN (Mahmood et al., 2019). The present work extends the understanding of the role of ATE in mediating the personality trait variables, namely NFA, PRP, and SLE, on the ENIN. Nevertheless, the ATE had no mediating effect on the correlation between RTP and ENIN. Additionally, ENE and UNA significantly affected ORC, while ENK had no significance. It was revealed that ORC exhibited no significant effect on students’ ENIN. Besides, ORC had no mediating effect on the relationships of ENE, UNA, and ENK with ENIN. These show that entrepreneurs, at times, can perceive opportunities after having the intention to become entrepreneurs.

In terms of practical implications, the study results offer insights into students’ ENIN and the factors that could motivate them to generate the ENIN. The government, universities, and other related institutions should support the growth of entrepreneurial activities in Malaysia. The Malaysian government may apply the study outcomes to devise and regulate policies in facilitating and reinforcing entrepreneurial activities (Mahmood et al., 2020). Entrepreneurial activities can boost economic growth and productive employment, whereby socio-economic issues such as income gap and unemployment may be effectively addressed by encouraging entrepreneurship (Bagheri, 2017).

The study findings demonstrated that students’ ATE had positively affected their ENIN. Therefore, universities and educational institutions should promote entrepreneurship by designing entrepreneurship courses, curriculums, and programmes to pique students’ interest in entrepreneurship as a career. Moreover, universities should provide a learning environment that is conducive, positively challenging, proactive, and able to increase students’ confidence to become entrepreneurs so that the students will have a positive ATE, thus boosting their ENIN. Similarly, this study revealed the significant positive effect of NFA, PRP, and SLE on ATE. Although this study had focussed on university students as the main subject, the outcomes may apply to students of other educational institutions in Malaysia, including secondary schools, mainly because nurturing entrepreneurial skills should start before students enter universities.

Most importantly, this study sheds light that entrepreneurship is a viable career path for university students who will soon graduate and join the workforce. This study highlights the best personality traits to develop for entrepreneurs, such as the NFA, PRP, and SLE. As such, students should gain exposure to external factors, including ENE and UNA, to strengthen their ability to perceive opportunities.

## Conclusion

The factors, such as misalignment of skills, lack of employment opportunities, an economic downturn, have prevented people’s ability to gain employment (Kim et al., 2018). The number of those unemployed in Malaysia is increasing, and there is a widening gap of incomes between the top 20% (T20) and bottom 40% (B40) groups of the Malaysian population (Mahmood et al., 2019). In particular, fresh graduates from universities without working experience will have difficulty securing a job in this competitive environment. Entrepreneurship drives economic prosperity, employment, and innovation cycle (Hassan et al., 2020). Hence, people have begun to choose entrepreneurship as their career path (Bagheri, 2017).

The study emphasises the significance of the ATE—generated from the NFA, PRP, and SLE—as a predictor of ENIN. The outcomes are in line with the TPB framework adopted in this study. Nonetheless, RTP displayed no significant effect on ATE and ENIN. Additionally, it was revealed that ORC was significantly affected by ENE and UNA but not by ENK. These findings demonstrate that ORC is not a predictor of ENIN, as some may intend to become entrepreneurs even before they recognise the opportunities.

The present study is not without limitations. In the current work-study, 391 university students from Malaysia were selected as respondents, and the online media platform was applied to disseminate the survey questionnaire. The questionnaire was not evenly distributed due to several factors stemming from the respondents’ demographic characteristics having slight variances. Hence, the study’s findings have good generalisation to university students in Malaysia with varying characteristics like family income, parent engagement in an entrepreneurial venture, or professional education of the family. Having the limitation, future studies may look into the variances in respondents’ demographic characteristics as the Malaysian population is rich in diversity. Secondly, the study had examined a factor that has not been widely discussed; ORC, which appeared insignificant for ENIN. Besides, as the study had dismissed entrepreneurial behaviour, future endeavours may assess the entrepreneurial behaviour to determine intention’s impact on entrepreneurial behaviour. Lastly, the current work assumed an empirical quantitative research design associated with the specific limitation. We suggest that future research must be designed to expose and explore attitude formation fully and opportunity recognition instigating the ENIN qualitatively.

## Data Availability Statement

The original contributions presented in the study are included in the article/Supplementary Material, further inquiries can be directed to the corresponding author/s.

## Ethics Statement

Ethical review and approval was not required for the study on human participants in accordance with the local legislation and institutional requirements. The patients/participants provided their written informed consent to participate in this study.

## Author Contributions

KW, VN’d, UM and QY: methodology, data collection, data analysis, and writing—original draft. AA, AS and NH: conceptualisation, methodology, and writing—review and revision. All authors contributed to the article and approved the submitted version.

## Conflict of Interest

The authors declare that the research was conducted in the absence of any commercial or financial relationships that could be construed as a potential conflict of interest.

## Publisher’s Note

All claims expressed in this article are solely those of the authors and do not necessarily represent those of their affiliated organizations, or those of the publisher, the editors and the reviewers. Any product that may be evaluated in this article, or claim that may be made by its manufacturer, is not guaranteed or endorsed by the publisher.

## Footnotes

1
https://webpower.psychstat.org/wiki/tools/index

## Appendix

**Appendix 1:** Loadings and cross-loading.

Code	NFA	RTP	PRP	SLE	ENE	UNA	ENK	ATE	ORC	ENIN
NFA – Item 1	*0.722*	0.127	0.364	0.379	0.318	0.293	0.276	0.256	0.304	0.255
NFA – Item 2	*0.799*	0.084	0.550	0.489	0.392	0.286	0.345	0.324	0.199	0.362
NFA – Item 3	*0.825*	0.048	0.452	0.487	0.362	0.193	0.268	0.361	0.211	0.338
NFA – Item 4	*0.692*	0.033	0.473	0.376	0.318	0.407	0.152	0.241	0.262	0.266
RTP – Item 1	–0.065	*0.746*	0.063	0.078	0.143	0.097	0.211	0.085	0.176	0.058
RTP – Item 2	0.013	*0.623*	–0.025	0.070	0.151	0.241	0.155	0.016	0.299	0.021
RTP – Item 3	0.076	*0.731*	0.032	0.125	0.200	0.182	0.304	0.057	0.273	0.086
RTP – Item 4	0.155	*0.909*	0.190	0.222	0.238	0.222	0.351	0.170	0.241	0.189
RTP – Item 5	0.053	*0.655*	0.023	0.090	0.165	0.285	0.180	0.020	0.294	0.001
PRP – Item 1	0.418	0.007	*0.681*	0.421	0.296	0.301	0.203	0.262	0.213	0.236
PRP – Item 2	0.405	0.190	*0.758*	0.544	0.352	0.180	0.449	0.361	0.188	0.403
PRP – Item 3	0.467	0.085	*0.782*	0.498	0.373	0.271	0.357	0.374	0.213	0.414
PRP – Item 4	0.460	0.132	*0.743*	0.513	0.403	0.228	0.305	0.322	0.201	0.372
PRP – Item 5	0.476	0.073	*0.698*	0.535	0.321	0.259	0.325	0.296	0.200	0.333
SLE – Item 1	0.437	0.146	0.469	*0.697*	0.353	0.376	0.260	0.295	0.313	0.306
SLE – Item 2	0.415	0.194	0.555	*0.775*	0.384	0.321	0.394	0.445	0.244	0.443
SLE – Item 3	0.428	0.216	0.518	*0.726*	0.345	0.261	0.440	0.274	0.311	0.304
SLE – Item 4	0.459	0.117	0.546	*0.789*	0.384	0.154	0.446	0.369	0.212	0.407
SLE – Item 5	0.464	0.159	0.534	*0.792*	0.368	0.193	0.438	0.315	0.241	0.396
SLE – Item 6	0.393	0.047	0.422	*0.654*	0.289	0.317	0.176	0.297	0.253	0.250
SLE – Item 7	0.365	0.101	0.464	*0.695*	0.259	0.191	0.239	0.299	0.190	0.296
ENE – Item 1	0.295	0.190	0.288	0.302	*0.735*	0.237	0.594	0.473	0.357	0.494
ENE – Item 2	0.377	0.135	0.346	0.355	*0.754*	0.281	0.360	0.444	0.330	0.425
ENE – Item 3	0.343	0.117	0.411	0.379	*0.671*	0.209	0.355	0.671	0.252	0.610
ENE – Item 4	0.332	0.249	0.371	0.344	*0.751*	0.381	0.394	0.338	0.408	0.359
UNA – Item 1	0.341	0.180	0.339	0.303	0.376	*0.735*	0.271	0.196	0.426	0.181
UNA – Item 2	0.335	0.120	0.272	0.336	0.344	*0.743*	0.208	0.194	0.346	0.145
UNA – Item 3	0.273	0.160	0.226	0.286	0.298	*0.781*	0.236	0.185	0.405	0.126
UNA – Item 4	0.323	0.165	0.276	0.296	0.294	*0.790*	0.146	0.205	0.411	0.159
UNA – Item 5	0.179	0.199	0.159	0.150	0.158	*0.600*	0.110	0.099	0.331	0.078
UNA – Item 6	0.152	0.202	0.165	0.153	0.221	*0.723*	0.152	0.128	0.376	0.094
ENK – Item 1	0.300	0.239	0.454	0.442	0.464	0.214	*0.782*	0.457	0.213	0.534
ENK – Item 2	0.292	0.353	0.361	0.371	0.526	0.255	*0.902*	0.329	0.375	0.390
ENK – Item 3	0.314	0.304	0.396	0.434	0.522	0.192	*0.879*	0.432	0.274	0.508
ATE – Item 1	0.350	0.138	0.422	0.447	0.560	0.150	0.473	*0.891*	0.226	0.772
ATE – Item 2	0.344	0.130	0.403	0.427	0.574	0.209	0.393	*0.907*	0.235	0.689
ATE – Item 3	0.335	0.126	0.415	0.371	0.556	0.183	0.428	*0.916*	0.253	0.713
ATE – Item 4	0.378	0.154	0.383	0.431	0.549	0.229	0.398	*0.832*	0.291	0.629
ATE – Item 5	0.309	0.058	0.297	0.291	0.496	0.260	0.252	*0.789*	0.260	0.579
ORC – Item 1	0.141	0.330	0.161	0.237	0.314	0.340	0.365	0.164	*0.678*	0.153
ORC – Item 2	0.183	0.231	0.123	0.150	0.252	0.366	0.175	0.092	*0.717*	0.017
ORC – Item 3	0.284	0.189	0.219	0.285	0.401	0.393	0.245	0.308	*0.792*	0.205
ORC – Item 4	0.272	0.130	0.268	0.284	0.382	0.428	0.233	0.236	*0.731*	0.276
ENIN – Item 1	0.360	0.141	0.409	0.425	0.585	0.186	0.485	0.656	0.173	*0.842*
ENIN – Item 2	0.343	0.099	0.407	0.391	0.559	0.135	0.476	0.776	0.198	*0.910*
ENIN – Item 3	0.357	0.116	0.454	0.452	0.544	0.196	0.411	0.727	0.255	*0.909*
ENIN – Item 4	0.355	0.092	0.443	0.425	0.516	0.137	0.419	0.693	0.191	*0.909*
ENIN – Item 5	0.287	0.274	0.359	0.336	0.540	0.177	0.683	0.383	0.277	*0.594*
ENIN – Item 6	0.370	0.122	0.420	0.408	0.488	0.116	0.389	0.673	0.187	*0.881*

*NFA, need for achievement; RTP, risk-taking propensity; PRP, proactive personality; SLE, self-efficacy; ENE, entrepreneurship education; UNA, uncertainty avoidance; ENK, entrepreneurial knowledge; ATE, attitude towards entrepreneurship; ORC, opportunity recognition competency; ENIN, entrepreneurial intention. The Italic values in the matrix above are the item loadings and others are cross-loadings. Source: Author’s data analysis.*
